# Supplementary material for: Prevalence, Molecular Detection, and Antimicrobial Resistance of Salmonella Isolates from Poultry Farms across Central Ethiopia: A Cross-Sectional Study in Urban and Peri-Urban Areas
Source: Microorganisms. 2024 Apr 10;12(4):767. doi: 10.3390/microorganisms12040767 (PMC11051739; doi:10.3390/microorganisms12040767)
Supplement: Supplementary file 1 [file microorganisms-12-00767-s001.zip › microorganisms-2912798-supplementary.pdf]

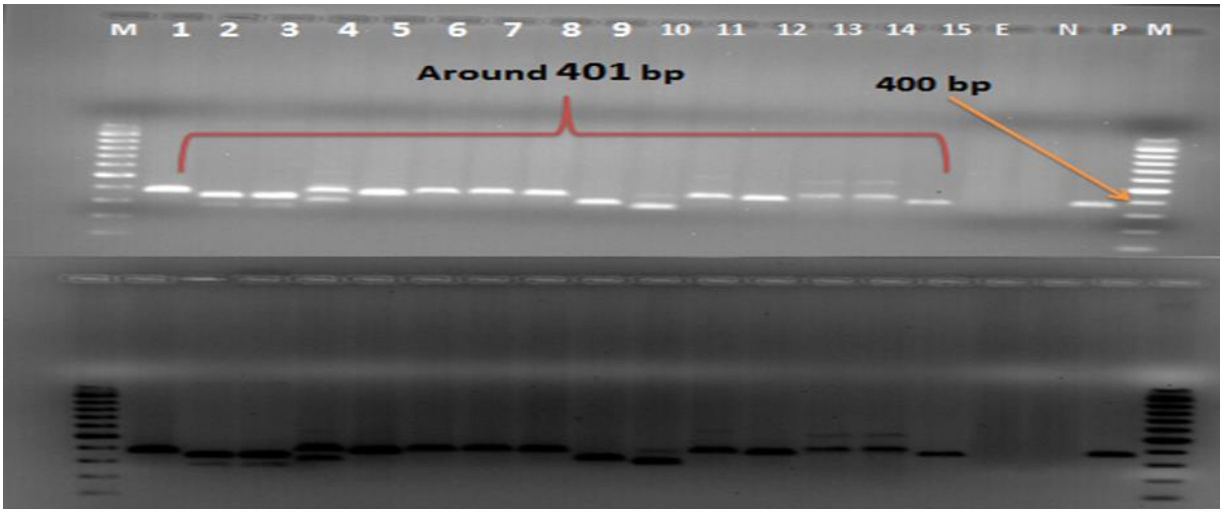

M: molecular marker (100 bp); Lane 1-15: *Salmonella* Typhimurium positive for *Spy* gene; Lane 16: extraction control (devoid of template DNA); Lane 17 negative control (nuclease-free water) and Lane 18: positive control (*Salmonella* Typhimurium positive isolate).

Figure S1. Electrophoresis of *Spy* gene for *Salmonella* Typhimurium isolates in 1.5 % agarose gel.

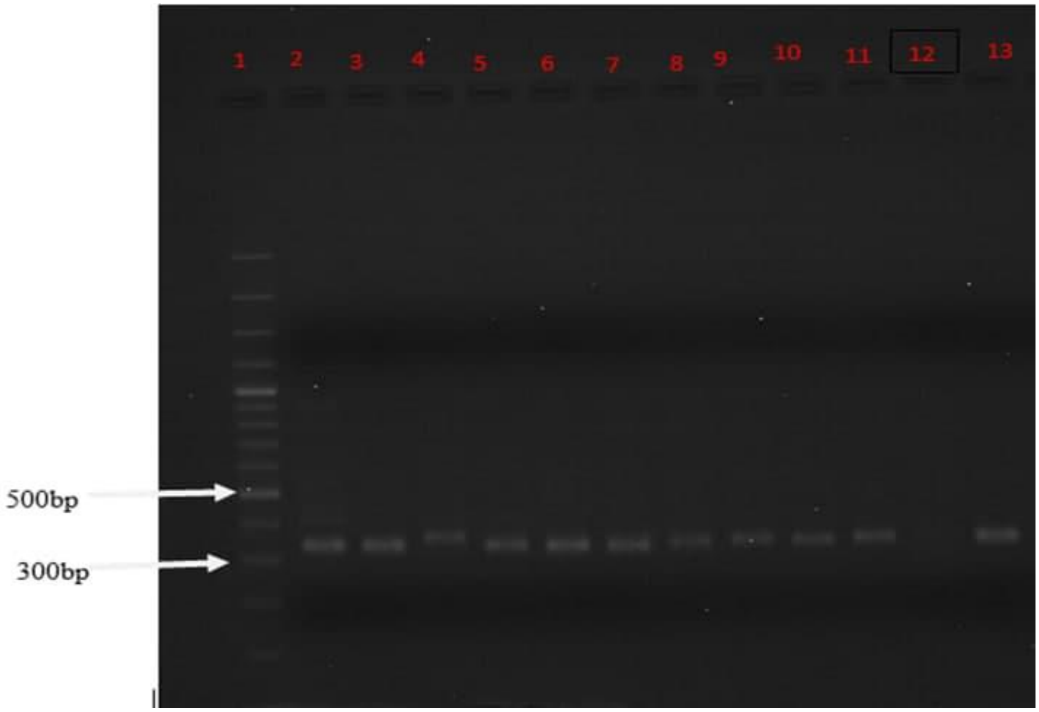

Lane 1:1 kb molecular ladder, Lane 2- 11 (Lane 2-11) were positive for *SdfI* gene that amplified at 304bp, Lane 12: negative control and Lane 13: positive controls were negative and positive control, respectively.

Figure S2. Conventional PCR for *Salmonella* Enteritidis.
